# Improving the Efficiency of Multistep Short-Term Electricity Load Forecasting via R-CNN with ML-LSTM

**DOI:** 10.3390/s22186913

**Published:** 2022-09-13

**Authors:** Mohammed F. Alsharekh, Shabana Habib, Deshinta Arrova Dewi, Waleed Albattah, Muhammad Islam, Saleh Albahli

**Affiliations:** 1 Department of Electrical Engineering, Unaizah College of Engineering, Qassim University, Unaizah 56452, Saudi Arabia; 2 System Control Processing Research Group, Qassim University, Buraydah 51452, Saudi Arabia; 3 Department of Information Technology, College of Computer, Qassim University, Buraydah 51452, Saudi Arabia; 4 Faculty of Data Science and Information Technology, INTI International University, Nilai 71800, Negeri Sembilan, Malaysia; 5 Department of Electrical Engineering, College of Engineering and Information Technology, Onaizah Colleges, Onaizah 56447, Saudi Arabia

**Keywords:** electricity load forecasting, residual CNN, ML-LSTM, CNN-LSTM, electricity consumption

## Abstract

Multistep power consumption forecasting is smart grid electricity management’s most decisive problem. Moreover, it is vital to develop operational strategies for electricity management systems in smart cities for commercial and residential users. However, an efficient electricity load forecasting model is required for accurate electric power management in an intelligent grid, leading to customer financial benefits. In this article, we develop an innovative framework for short-term electricity load forecasting, which includes two significant phases: data cleaning and a Residual Convolutional Neural Network (R-CNN) with multilayered Long Short-Term Memory (ML-LSTM) architecture. Data preprocessing strategies are applied in the first phase over raw data. A deep R-CNN architecture is developed in the second phase to extract essential features from the refined electricity consumption data. The output of R-CNN layers is fed into the ML-LSTM network to learn the sequence information, and finally, fully connected layers are used for the forecasting. The proposed model is evaluated over residential IHEPC and commercial PJM datasets and extensively decreases the error rates compared to baseline models.

## 1. Introduction

Electricity load forecasting predicts future load based on single or multiple features or parameters. Features could be of multiple types, such as an ongoing month, hour, weather situation, electricity costs, economic conditions, geographical circumstances, etc. [1]. Electricity load forecasting is significantly increasing due to the development and extension of the energy market, as they endorsed trading electricity for each hour. Profitable market interactions could be enabled by accurate load forecasting [2] and leakage current prediction [3], which helps power firms guarantee electricity stability and decrease electricity wastage [4]. The electricity load prediction is handled through short-term electricity load forecasting [5], which is particularly significant due to smart grid development [6].

The United States Energy Information Administration stated that from 2015–40 the increase in power consumption would be boosted up to 28% [7], while the International Energy Agency stated that buildings and building construction account for approximately 36% of the world’s total energy consumption. Stimulating building energy efficiency is vital in the low carbon economy [8,9]. Accurate energy consumption forecasting is indispensable for buildings’ energy-saving design and renovation. The scrutiny of dissimilarity among the energy consumption forecasting and the experimented data can also provide a foundation for building operation monitoring. It can also provide a source for energy peak regulation for large buildings [10,11]. In addition, some large-scale buildings are essential to secure public resource supply in an area. Thus, accurate energy consumption forecasting can sustain the resident energy allocation sectors.

The following few important points could establish accurate forecasting models of consumption. First, it is crucial to precisely recognize the parameters that contain robust effects on a state’s consumption and add these indicators to the prediction model. Furthermore, choosing an appropriate modeling procedure is also a significant point. The input and output variables contain such a nonlinear relationship that it is very challenging to express them mathematically. Principles to advance the prediction performance develop conspicuously nowadays, instead of hypothetical principles in the model selection. In the end, the vital point is that the methodology must be capable of producing effective forecasting results.

Reviewing the literature in recent years, machine learning models are increasingly used by scholars to forecast the short-term energy consumption of buildings. Additionally, most scholars are adopting hybrid models due to their effective performance compared to single models. However, energy consumption in buildings is exaggerated by various factors and is extremely nonlinear. Thus, this area is still worthy and commendable to be studied and explored further. This research work contributes the following points to the current literature.

- The collected benchmark datasets contain a lot of missing values and outliers, which occur due to defaulted meters, weather conditions, and abnormal customer consumption. These abnormalities and redundancies in datasets lead the forecasting network to ambiguous predictions. To resolve this problem, we performed data preprocessing strategies, including outlier removal via the three sigma rules of thumb algorithm, missing value via NAN interpolation method, and the normalization of the data using the MinMax scaler.
- We present a deep R-CNN integrated with ML-LSTM for power forecasting using real power consumption data. The motivation behind R-CNN with ML-LSTM is to extract patterns and time-varied information from the input data for effective forecasting.
- The proposed model results in the lowest error rates of MAE, MSE, RMSE, and MAPE and the highest R^2^ compared to recent literature. For the IHEPC dataset, the proposed model achieved 0.0447, 0.0132, 0.002, 0.9759, and 1.024 for RMSE, MAE, MSE, R^2^, and MAPE, respectively, over the hourly IHEPC dataset while these values are 0.0447, 0.0132, 0.002, 0.9759, and 1.024 over the IHEPC daily dataset. For the PJM dataset, the proposed model achieved 0.0223, 0.0163, 0.0005, 0.9907, 0.5504 for RMSE, MAE, MSE, R^2^, and MAPE, respectively. The lowest error metrics indicated the supremacy of the proposed model over state-of-the-art methods.

## 2. Literature Review

Short-term load forecasting is a current research area, and numerous studies have been conducted in the literature. These studies are mainly divided into four categories based on the learning algorithm: physical, persistence, artificial intelligence (AI), and statistical. The persistence model can predict future time series data behavior like electricity consumption or forecasting but failed for several hour ahead predictions [12]. Therefore, persistence models are not decisive for electricity forecasting. Physical models are based on mathematical expressions that consider meteorological and historical data. N. Mohan et al. [13] present a dynamic empirical model for short-term electricity forecasting based on a physical model. These models are also unreliable for electricity forecasting due to the high memory, and computational space required [12]. As compared to physical models, statistical models are less computationally expensive [14] and are typically based on autoregressive methods, i.e., GARCH [15], ARIMA [16], and linear regression methods. These models are based on linear data, while electricity consumption prediction or load forecasting is a nonlinear and complex problem. As presented in [12], the GARCH model can capture uncertainty but has limited capability to capture non-stationary and nonlinear characteristics of electricity consumption data. Several studies based on linear regression have also been developed in previous studies. For instance, N. Fumo et al. proposed a linear and multiple regression model for electricity load forecasting [17]. Similarly, [18,19] developed multi-regression-based electricity load forecasting models. Statistical-based models cannot capture uncertainty patterns directly but use other techniques, as presented in [20], to reduce uncertainty. AI methods can learn nonlinear complex data, divided into shallow and deep structure methods (DSM). The shallow-based methods like SVM [21], ANN [22], wavelet neural networks [23], random forest [24], and ELM [25] performed poorly, except for feature mining. Thus, improving the performance of these models additionally needs feature extraction and selection methods, remaining a challenging problem [20,26].

The AI methods are further categorized into machine learning and deep learning. In machine learning, several studies have been conducted in the literature for electricity load forecasting. Chen et al. used SVR for power consumption forecasting using electricity consumption and temperature data [27]. Similarly, in [28], an SVR-based hybrid electricity forecasting model was developed. In this study, the SVR model was integrated with adjusting the seasonal index and optimization algorithm fruity fly for better performance. Zhong et al. [29] proposed a vector field base SVR model for energy prediction and transformed the nonlinearity of the feature space into linearity to address the concern of the nonlinearity of the model to the input data. C. Li et al. conducted a study based on Random Forest Regression (RFR). First, the features are extracted in the frequency domain using fast Fourier transformation and then used RFR model for simulation and prediction [30]. Similarly, several deep learning models are developed for short-term electricity load forecasting. W. Kong et al. used LSTM recurrent network for electricity forecasting [31]. Another study [32] proposed bidirectional LSTM with an attention mechanism and rolling update technique for electricity forecasting. A recurrent neural network (RNN), based on a pooling mechanism, is developed in [20] to address the concern of overfitting.

Features extraction methods include different regular patterns, spectral analysis, and noise. However, these methods reduce the accuracy for meter level load [20] and these methods reduce the accuracy due to the low proportion of regular patterns in electricity data. DSM, also called deep neural networks (DNNs), addressed the challenges in shallow-based methods. DNN-based models are based on multi-layer processing and learn hierarchical features from input data. For sequence and pattern learning, LSTM [20,26] and CNN [33] are the most powerful architecture recently proposed. The long-tailed dependencies in raw time series data LSTM cannot capture it [34].

Similarly, CNN networks cannot learn temporal features of power consumption. Therefore, hybrid models are developed to forecast power consumption effectively. T. Y. Kim et al. combine CNN with LSTM for power consumption prediction [35], while another study presented in [36] used CNN with bidirectional LSTM. ZA Khan et al. developed a CNN-LSTM-autoencoder-based model to predict short-term load forecasting [37]. Furthermore, CNN with a GRU-based model is developed in [38]. These models address the problems in DNN networks, but the prediction accuracy is unreliable for real-time implementation. Therefore, we developed a two-stage framework for short-term electricity load forecasting in this work. In the first stage, the raw electricity consumption data collected from a residential house is preprocessed to remove missing values, outliers, etc. This refined data is then fed to our newly proposed R-CNN with ML-LSTM architecture to address the concerns of DNNs and improve the forecasting performance for effective power management.

## 3. Proposed Method

**Figure 1:**
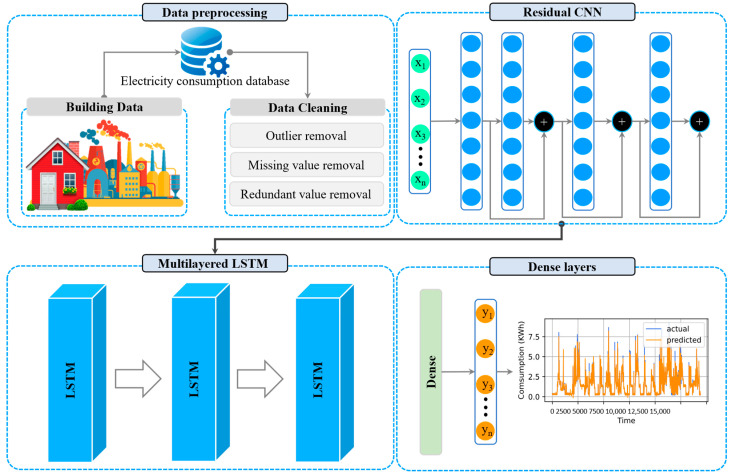
Proposed framework for electricity consumption forecasting.

The overall architecture of R-CNN with ML-LSTM is shown in Figure 1 for short-term electricity load forecasting. A two-stage framework is presented, which includes data preprocessing and proposed R-CNN with ML-LSTM architecture. Data preprocessing includes filling missing values removing outliers, and normalizing the data for efficient training. The second step comprises R-CNN with ML-LSTM architecture, where R-CNN is employed for pattern learning. At the same time, the ML-LSTM layers are incorporated to learn the sequential information of electricity consumption data. Each step of the proposed framework is further explained in the following sub-sections.

### 3.1. Data Preprocessing

Smart meter sensors-based data generation contains outliers and missing values for several reasons, such as meter faults, weather conditions, unmanageable supply, storage issues, etc. [39], and must be preprocessed before training. Herein, we apply a unique preprocessing step. For evaluating the proposed method, we used IHEC Dataset, which includes the above-mentioned erroneous values. In addition, the performance is evaluated over the PJM benchmark dataset. To remove the outlier values in the dataset, we used three sigma rules [40] of thumb according to Equation (1).

(1) $$Ƒ\left(D_{i}\right)=\left\{\begin{array}{cc} AVG\left(d\right)+2.STD\left(d\right), & if\;d_{i\;}>avg\left(d\right)+std\left(d\right)\\ d_{i}, & otherwise \end{array}\right.$$

where $d_{i}$ is a vector or superset of A representing a value for power consumption in duration, i.e., minute, hour, day, etc. At the same time, $AVG\left(d\right)$ is the average of D, and $STD\left(D\right)$ represents the standard deviation of the D. A recovering interpolation method is used as presented in Equation (2) for missing values.

(2) $$Ƒ\left(D_{i}\right)=\left\{\begin{array}{cc} \frac{d_{i-1}+d_{i+1}}{2}, & d_{i}\in nan,d_{i-1},d_{i+1}\notin nan\\ 0, & d_{i}\in nan,d_{i-1}\;or\;d_{i+1}\in nan\\ d_{i}, & d_{i}\notin nan \end{array}\right.$$

**Figure 2:**
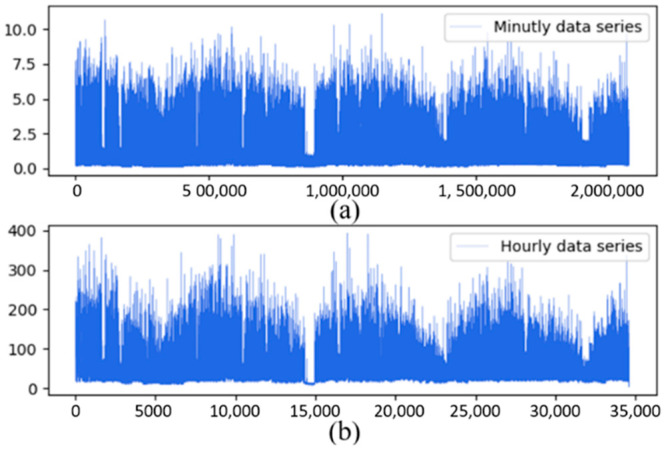
Data down sampling where “(**a**)” shows actual minute data and “(**b**)” shows hourly down-sampled data.

If $d_{i}$ is missing or null, we placed it as a NAN. The IHEPC dataset was recorded in a one minute resolution, while the PJM dataset was recorded in a one hour resolution. The IHEPC dataset is down sampled for daily load forecasting into hourly resolutions. The input datasets are resampled into low resolution (from minutes to hours) in downsampling. The IHEPC dataset includes 2,075,259 recodes, down-sampled into 34,588 records for daily load forecasting, as shown in Figure 2.

After data cleaning, we apply the data transformation technique to transform the cleaned data into a specific format more suitable for effective training. First, we use the power transformation technique to remove shifts and transform the data into more Gaussian-like. The power transformation includes Box–Cox [41] and Yeo–Johnson [42]. The Box–Cox is sensitive to negative values, while Yeo–Johnson can support both negative and positive values. In this work, we used the Box–Cox technique for power transformation to remove a shift from the electricity data distribution. This work uses univariate electricity load forecasting datasets, so the Box–Cox transformation for a single parameter is shown in Equation (3).

(3) $$d_{i}^{\left(\lambda \right)}=\left\{\begin{array}{cc} \frac{d_{i}^{\left(\lambda \right)}-1}{\lambda }, & if\;D\neq 0\\ Ind, & if\;D=0 \end{array}\right.$$

Finally, the min–max data normalization technique converts the data into a specific range because deep learning networks are sensitive to diverse data. The equation of min–max normalization is shown in Equation (4).

(4) $$D_{i}=\frac{d–d_{min}}{d_{max}–d_{min}}$$

where d is the actual data, while $d_{min}$ is the minimum and $d_{max}$ is the maximum values in the dataset.

### 3.2. R-CNN with ML-LSTM

The proposed architecture integrates R-CNN with ML-LSTM for power load forecasting. R-CNN and ML-LSTM can store the complex fluctuating trends and extract complicated features for electricity load forecasting. First, the R-CNN layers extract patterns, which are then passed to ML-LSTM as input for learning. CNN is a well know deep learning architecture consisting of four types of layers: convolutional, pooling, fully connected, and regression [43,44]. The convolutional layers include multiple convolution filters, which perform a convolutional operation between convolutional neuron weights and the input volume-connected region, which generates a feature map [45,46]. The basic equation of the convolutional layer operation is shown in Equation (5).

(5) $${𝒞}_{m,n,o}^{ȴ}=Ƒ({\left(W_{o}^{ȴ}\right)}^{t}\;X_{m,n}^{ȴ}+{Ƅ}_{k}^{ȴ}$$

where ${Ƅ}_{k}^{ȴ}$ is the bias of *o*th convolution filter in the *l*th layer and $X_{m,n}^{ȴ}$ demonstrates the location and activation function Ƒ(). In the convolution operation, the weights $W_{o}^{ȴ}$ must be shared with the overall input region, known as weight sharing. During model building, the weight sharing significantly decreases the cost of calculation time and training parameter numbers. After convolution, the pooling operation is performed. The pooling layer reduces feature map resolution for input feature aggregation [47,48]. The output of the pooling layer is shown in Equation (6).

(6) $${Ƥ}_{m,n,o}^{ȴ}=\mathrm{Pool}\left(p_{i,j,o}^{ȴ}\right)$$

where $\left(i,j\right)$ ε $P_{m,n}$, ε $P_{m,n}$ is the region of location of i,j. CNN has three types of pooling layers: max, min, and average poling. The CNN’s general network comprises several convolutional and pooling layers. Before the regression, the fully connected layers are typically set where every neuron in the previous layer is connected to every other in the next layer. The main purpose of the fully connected layer is to represent the learned feature distribution to a single space for high-level reasoning. The regression layer is the final output of the CNN model.

Due to the strong feature extraction ability, CNN architectures are extensively applied for image classification, video classification, time series, etc. Similarly, in time-series forecasting, these models are used for traffic [49,50], renewables [51], election prediction [52], and power forecasting [53]. Recent studies of image classification show the crucial performance of CNNs. As the network depth increases to a certain level, the degradation problem occurs in which the model performance is saturated. The experimentation shows that saturation is an optimization problem that is not caused by overfitting. To address the degradation concern, R-CNN architecture has been developed [54]. The conventional CNN learns the data in a linear mechanism, i.e., a direct function $Ƒ\left(a\right)$, but the R-CNN learns it differently, defined as $H\left(a\right)=Ƒ\left(a\right)+a$.

The ResNet solves the degradation problem and performs satisfactory results over image recognition data, but electricity consumption is time series sequential data. The CNN architecture cannot learn the sequential features of power consumption data. Therefore, the R-CNN with ML-LSTM architecture is developed in this research study for future electricity load forecasting. The R-CNN layers extract spatial information from electricity consumption data. The extracted features of R-CNN are then fed to ML-LSTM as input for temporal learning.

The output of R-CNN is then forwarded to ML-LSTM architecture, that is responsible for storing time information. The ML-LSTM maintains long-term memory by merging its units to update the earlier hidden state, aiming to understand temporal relationships in the sequence. The three gates unit’s mechanism is incorporated to determine each memory unit state through multiplication operations. The input gate, output gate, and forget gate represent each gate unit in the LSTM. The memory cells are updated with an activation. The operation of each gate in the LSTM can be shown in Equations (7)–(9), and the output of each gate is represented by i, f, and o notation, while ∂ is the activation function, w represents the weight, and b is the bias.

(7) $$i_{t}=\partial (W_{i}\left[h_{𝓽-1},\;{𝔁}_{𝓽}\right]+b_{i}$$

(8) $$f_{t}=\partial (W_{f}\left[h_{𝓽-1},\;{𝔁}_{𝓽}\right]+b_{f}$$

(9) $$o_{t}=\partial (W_{o}\left[h_{𝓽-1},\;{𝔁}_{𝓽}\right]+b_{o}$$

### 3.3. Architecture Design

The proposed R-CNN with ML-LSTM is based on three types of layers: R-CNN, ML-LSTM, and fully connected layers. The kernel size, filter numbers, and strides are adjustable in R-CNN layers according to the model’s performance. Many learning speeds, changes, and performance can happen by adjusting these parameters varying on the input data [55]. We can confirm the performance change by increasing or decreasing these parameters. We used a different kernel size in each layer to minimize the loss of temporal information. The data pass through the residual R-CNN layer, followed by the pooling layer for pattern learning. The output is then fed to the ML-LSTM for sequence learning, which is then forwarded to fully connected (FC) layers for final forecasting. Table 1 shows the layer type, the kernel’s size, and R-CNN parameters with the ML-LSTM network.

## 4. Results

The experimental setup, evaluation metrics, dataset, performance assessment over hourly data, and performance assessment over daily data of R-CNN with the ML-LSTM model are briefly discussed in the following section.

### 4.1. Experimental Setup

To validate the effectiveness of the proposed approach, the IHEPC dataset is used to implement comprehensive experiments. The R-CNN with ML-LSTM is trained over an Intel-Core-i7 CPU having 32GB RAM and GEFORCE-GTX-2060-GPU in Windows 10. The implementation was performed in Python 3.5 using the Keras framework.

### 4.2. Evaluation Metrics

The model performance is evaluated on mean square error (MSE), mean absolute error (MAE), root mean square error (RMSE), coefficient of determination ($R^{2})$, and mean absolute percentage error (MAPE) metrics. MAE computes the closeness between actual and forecasted values, MSE calculates square error, RMSE is the square root of MSE, $R^{2}$ exhibits model fitting effect ranging from 0 to 1 where closer to 1 indicates best prediction performance, and MAPE is the absolute ratio error for all samples. The mathematical equations of each metric are demonstrated in Equations (10)–(12) Where $y_{i}$ is the actual power consumption value and ${\hat{y}}_{i}$ is the forecasted value.

(10) $$MAE=\frac{\sum_{i=1}^{n}\left|y_{i}-{\hat{y}}_{i}\right|}{n}$$

(11) $$MSE=\frac{\sum_{i=1}^{n}{\left(y_{i}-{\hat{y}}_{i}\right)}^{2}}{n}$$

(12) $$RMSE=\sqrt{\frac{\sum_{i=1}^{n}{\left(y_{i}-{\hat{y}}_{i}\right)}^{2}}{n}}$$

(13) $$R^{2}=1-\frac{\sum_{i=1}^{n}{\left(y_{i}-{\hat{y}}_{i}\right)}^{2}}{\sum_{i=1}^{n}{\left(y_{i}-y_{i}\right)}^{2}}$$

(14) $$MAPE=\frac{1}{n}\overset{n}{\underset{i=1}{\sum }}\left|\frac{y_{i}-{\hat{y}}_{i}}{y_{i}}\right|$$

### 4.3. Datasets

The R-CNN with ML-LSTM model is evaluated over the UCI repository’s IHEPC and PJM datasets. The IHEPC comprises nine attributes: date and time variables, active and reactive power, voltage, intensity, and three submetering variables. Description of IHEPC attributes and their unit are shown in Table 2. The IHEPC dataset was collected from a residential building in France between the period of 2006 to 2010. PJM (Pennsylvania-New Jersey-Maryland) is a regional transmission society that operates the eastern electricity grid of the US. The PJM transmits the electricity to several US regions, including Maryland, Michigan, Delaware, etc. The power consumption data are stored on PJM’s official website, which is recorded in one-hour resolution in megawatts.

### 4.4. Comparative Analysis

The performance of R-CNN with the ML-LSTM model is compared to other models over residential electricity consumption IHEPC and regional electricity forecasting PJM datasets. The performance of the proposed model over these datasets and its comparison with other models are clarified in the subsequent sections.

## 5. Evaluation of IHEPC Dataset

**Figure 3:**
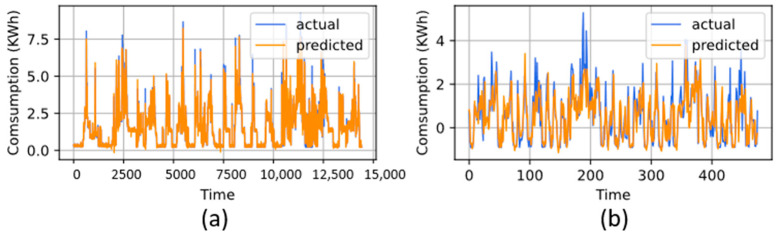
Visual results of R-CNN with ML-LSTM for IHEPC (**a**) hourly and (**b**) daily.

The supremacy of the proposed model over the IHEPC dataset is evaluated on hourly and daily load forecasting, whereas the R-CNN with ML-LSTM achieved satisfactory results. The performance of the test dataset for daily and hourly load forecasting is shown in Figure 3a,b. The comparison with other baseline methods for hourly load forecasting is presented in Table 3. For hourly load forecasting, the results are compared with linear regression [35], ANN [56], CNN [37], CNN-LSTM [35], CNN-BDLSTM [36], CNNLSTM-autoencoder [37], SE-AE [57], GRU [58], FCRBM [59], CNNESN [60], residual CNN stacked LSTM [61], CNN-BiGRU [62], CNN-GRU [63], STLF-Net [64], residual GRU [65] and Khan et al. [66]. Compared to all these studies, R-CNN with ML-LSTM achieved lower error rates of 0.0325, 0.0144, 0.0011, 0.9841, and 1.024 for RMSE, MAE, MSE, R^2^, and MAPE, respectively.

The performance of the proposed model over the hourly resolution of data also secured the lowest error rate compared to the baseline model. For a precedent, the performance over the daily resolution of data is compared with regression [35], CNN [37], LSTM [35], CNN-LSTM [35], and FCRBM [59], where the details results of each study are given in Table 4. Comparatively, the R-CNN with ML-LSTM model also reduces the error rates over the daily dataset and achieved 0.0447, 0.0132, 0.002, 0.9759, and 2.457 for RMSE, MAE, MSE, R^2^, and MAPE, respectively, for daily load forecasting.

## 6. Evaluation of the PJM Dataset

**Figure 4:**
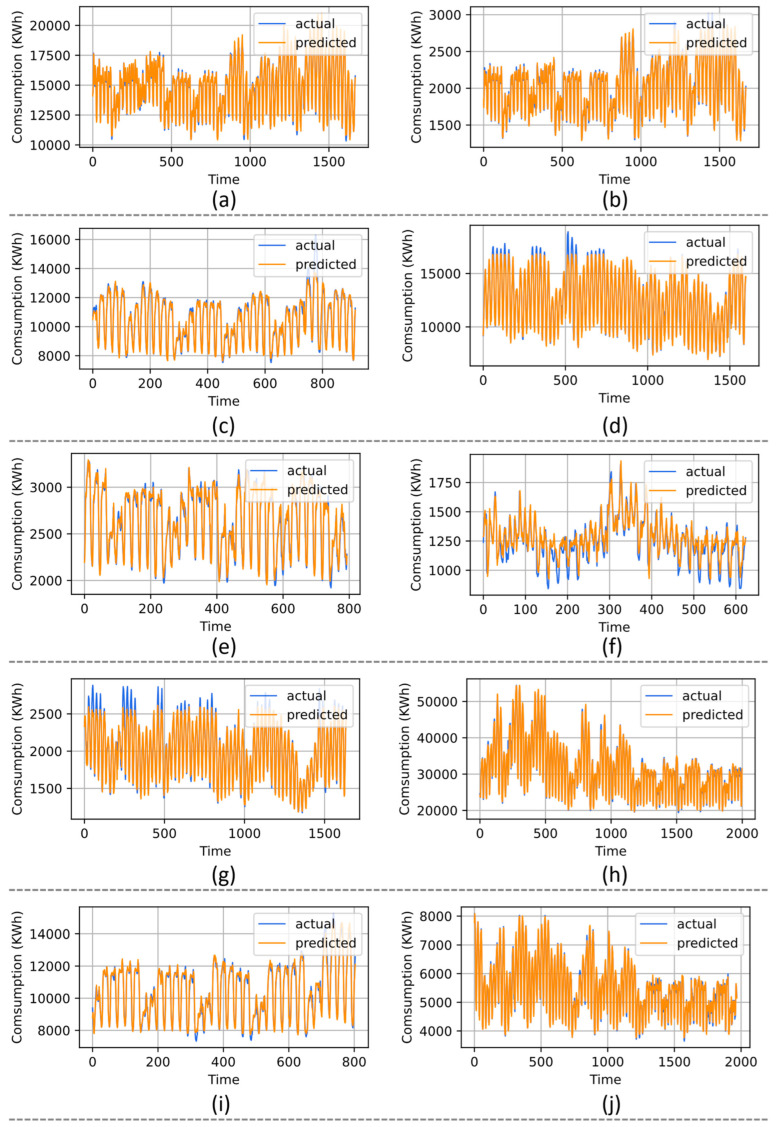
Visual results of the proposed model over IHEPC dataset (**a**) AEP, (**b**) DAYTON, (**c**) COMED, (**d**) DOM, (**e**) DEOK, (**f**) EKPC, (**g**) DUQ, (**h**) PJME, (**i**) NI, and (**j**) PJMW dataset.

The superiority of R-CNN with ML-LSTM is also evaluated over several PJM datasets for daily load forecasting. The PJM benchmark includes 14 datasets of electricity load forecasting, while for the experimentation, we chose 10 datasets from the PJM as selected by [61]. In the literature, we found the comparison for 10 regions dataset, which is demonstrated in Table 5, where the proposed model acquired the lowest error rate for each dataset. The performance of R-CNN with ML-LSTM is compared with Mujeeb et al. [67], Gao et al. [68], Chou et al. [69], Khan et al. [61], and Han et al. [58]. The R-CNN with ML-LSTM secures the lower error metrics for all datasets, where the details are given in Table 5, while the prediction results for all datasets in the PJM region are shown in Figure 4.

## 7. Conclusions

A two-phase framework is proposed in this work for power load forecasting. Data cleaning is the first phase of our framework where data preprocessing strategies are applied over raw data to make it clean for effective training. Secondly, a deep R-CNN with ML-LSTM architecture is developed where the R-CNN learns patterns from electricity data, and the outputs are fed to ML-LSTM layers. Electricity consumption comprises time series data that include spatial and temporal features. The R-CNN layers extract spatial features in this work, while the ML-LSTM architecture is incorporated for sequence learning. The proposed model was tested over residential and commercial benchmark datasets and conducted with satisfactory results. For residential power consumption forecasting, IHEPC data was used, while the PJM dataset was used for commercial evaluation. The experiments are performed for daily and hourly power consumption forecasting and extensively decrease the error rates. In the future, the proposed model will be tested over medium-term and long-term electricity load forecasting. In addition, we will integrate environmental sensor data that help to predict future electricity consumption. Furthermore, we also intend to investigate the performance of the R-CNN and ML-LSTM in other prediction domains such as fault prediction, renewable power generation prediction, and traffic flow prediction.

## Figures and Tables

**Table 1:** The internal architecture of R-CNN and ML-LSTM.

Layer	Filter-Size	Kernel-Size	Layer-Parameter
Input		-	-
Convolutional (conv)_1	32	7	10,816
conv_2	32	5	20,544
Add [conv_1, conv_2]		-	-
conv_3	64	3	12,352
Add [conv_2, conv_3]		-	-
Convolutional_4	128	1	4160
Add [conv_3, conv_4]		-	-
LSTM	100	-	66,000
LSTM	100	-	80,400
LSTM	100	-	80,400
FC	128	-	12,928
FC	60	-	7740
**Total parameters**			**295,340**

**Table 2:** IHEPC dataset attributes and units.

Attributes	Description	Units
Date and time	Comprise the range of datetime values	dd/mm/yyyy and hh:mm:ss
Global active, reactive power and intensity	Minutely averaged Global active1, reactive Power2, and intensity3 values	kilowatt (Kw)1,2
Ampere (A)3		
Voltage	Minutely averaged voltage values	Volt(V)

**Table 3:** Performance comparison of R-CNN and ML-LSTM with state-of-the-art over hourly IHEPC dataset.

Method	RMSE	MAE	MSE	R^2^	MAPE
Linear regression [35]	0.6570	0.5022	0.4247	-	83.74
ANN [56]	1.15	1.08	-	-	-
CNN [37]	0.67	0.47	0.37	-	-
CNNLSTM [35]	0.595	0.3317	0.3549	-	32.83
CNN-BDLSTM [36]	0.565	0.346	0.319	-	29.10
CNNLSTM-autoencoder [37]	0.47	0.31	0.19	-	-
SE-AE [57]	-	0.395	0.384	-	-
GRU [58]	0.41	0.19	0.17	-	34.48
FCRBM [59]	0.666	-	-	−0.0925	
CNNESN [60]	0.0472	0.0266	0.0022	-	-
Residual CNN Stacked LSTM [61]	0.058	0.003	0.038	-	-
CNN-BiGRU [62]	0.42	0.29	0.18	-	-
CNN-GRU [63]	0.47	0.33	0.22	-	-
STLF-Net [64]	0.4386	0.2674	0.1924	-	36.24
Residual GRU [65]	0.4186	0.2635	0.1753	-	-
ESN-CNN [66]	0.2153	0.1137	0.0463	-	-
**Proposed**	**0.0325**	**0.0144**	**0.0011**	**0.9841**	**1.024**

**Table 4:** Performance comparison of R-CNN and ML-LSTM with state-of-the-art over daily IHEPC dataset.

Method	RMSE	MAE	MSE	R^2^	MAPE
Linear regression [35]	0.5026	0.3915	0.2526	-	52.69
CNN [37]	0.07	0.05	0.006	-	-
LSTM [35]	0.4905	0.4125	0.2406	-	3872
CNN-LSTM [35]	0.3221	0.2569	0.1037		37.83
FCRBM [59]	0.828	-	-	0.3304	
**Proposed**	**0.0447**	**0.0132**	**0.002**	**0.9795**	**2.457**

**Table 5:** Performance comparison of the proposed model with state-of-the-art forecasting methods over the PJM hourly dataset.

Dataset	Method	RMSE	MAE	MSE	R^2^	MAPE
AEP	Mujeeb et al. [67]	0.386	-	-	-	1.08
	Gao et al. [68]	0.49	-	-	-	1.14
	Han et al. [58]	0.054	-	-	-	-
	Khan et al. [61]	0.031	0.001	0.027	-	-
	**Proposed**	**0.0223**	**0.0163**	**0.0005**	**0.9907**	**0.5504**
DAYTON	Khan et al. [61]	0.046	0.033	0.002	-	-
	**Proposed**	**0.0206**	**0.0144**	**0.0004**	**0.9911**	**0.4982**
COMED	Khan et al. [61]	0.044	0.030	0.002	-	-
	**Proposed**	**0.0216**	**0.0131**	**0.0005**	**0.9906**	**0.5475**
DOM	Khan et al. [61]	0.057	0.039	0.003	-	-
	**Proposed**	**0.0212**	**0.0138**	**0.0005**	**0.9905**	**0.5987**
DEOK	Khan et al. [61]	0.053	0.036	0.003	-	-
	**Proposed**	**0.0174**	**0.0129**	**0.0003**	**0.9932**	**0.3974**
EKPC	Khan et al. [61]	0.055	0.034	0.003	-	-
	**Proposed**	**0.0274**	**0.0202**	**0.0008**	**0.9882**	**0.7965**
DUQ	Khan et al. [61]	0.054	0.041	0.003	-	-
	**Proposed**	**0.0430**	**0.0277**	**0.0009**	**0.9975**	**0.8234**
PJME	Khan et al. [61]	0.043	0.031	0.002	-	-
	**Proposed**	**0.0199**	**0.0128**	**0.0004**	**0.9913**	**0.4721**
NI	Khan et al. [61]	0.050	0.033	0.002	-	-
	**Proposed**	**0.0178**	**0.0129**	**0.0003**	**0.9930**	**0.3748**
PJMW	Khan et al. [61]	0.038	0.027	0.001	-	-
	**Proposed**	**0.0145**	**0.0102**	**0.0002**	**0.9949**	**0.2864**
